# Effect of honeydew honey addition on the water activity and water holding capacity of kefir in the context of its sensory acceptability

**DOI:** 10.1038/s41598-021-02424-7

**Published:** 2021-11-25

**Authors:** Paulina Bielska, Dorota Cais-Sokolińska, Joanna Teichert, Jakub Biegalski, Łukasz K. Kaczyński, Sylwia Chudy

**Affiliations:** Department of Dairy and Process Engineering, Faculty of Food Science and Nutrition, Poznań University of Life Sciences, Poznań, Poland

**Keywords:** Chemical engineering, Microbiology, Plant sciences

## Abstract

The aim of the research was to check how the addition of honeydew honey and various compositions of starter cultures affects the water holding capacity, water activity, color, syneresis and consistency of the obtained kefir in the context of its sensory acceptability. In this research, 2.5% and 5% (w/w) honeydew honey was added to the samples of model kefir (K) and commercial kefir (K13). Kefirs differed by the type of used starter cultures and conditions of production. The addition of honeydew honey to kefir resulted in increased water holding capacity and a reduction in water activity. Honeydew honey kefir was characterized by the following flavor: astringent, fruity, pungent and waxy. As the honey content increased, the taste and waxy flavor became sweeter. In the sensory assessment, the attributes of texture and mouthfeel, creaminess, density and firmness, do not change because of the honey amount or storage time of the samples. The use of different starter cultures in kefir production with the addition of honeydew honey impacted texture parameters, resulting in up to a 4.8-fold increased viscosity index.

## Introduction

The global food industry, in many cases, uses lactic acid bacteria (LAB) to produce various of products^1^. An example of such a product is kefir, which consists of at least 2.7% protein, 0.6% lactic acid, and less than 10% fat^2^. Kefir, is of great interest to consumers owing to its various functional properties, including antibacterial, antioxidant, antitumor and hypocholesterolemic properties^3^. In addition, it is known that regular consumption of kefir improves digestion and tolerance to lactose^4^. Kefir has been consumed for thousands of years and occupies a significant place among functional foods^2^. What's more, kefir can be the basis for various substrates, which allows the production of new functional beverages^5^. The pro-health benefits of kefir result from the content of bioactive compounds, as the fermentation process increases the content of vitamins, folic acid, calcium and amino acids^6^. The popularity of fermented milk is related not only to its health benefits but also to its taste (slightly sour) and aroma. To create a more sensory appealing product, additives are used, such as aromas and sweeteners^7,8^. However, honey can be a better natural additive than artificial flavorings^9^. Honey is a natural product containing mainly fructose and glucose, with a low pH of approx. 3.9. Such pH is making it compatible with many food products in terms of acidity^10^. The composition of honey also includes bioactive compounds, including flavonoids, phenolic compounds, carotenoid derivatives, organic acids, Maillard reaction products, ascorbic acid and other compounds with antioxidant properties^11^. Additionally, clinical trials have shown that incorporation of honey into milk improves the sleep status of patients with acute coronary syndrome^12^.

There are two main types of honey: blossom or floral honey and honeydew honey^13,14^. Honeydew honey is characterized by its stronger taste, greater antioxidant activity, and higher concentrations of oligosaccharides than floral honey^14^. In addition, honeydew honey’s health benefits include improved circulation and dilation of coronary vessels, which regulates the workings of the heart. Narrowing of arteries occurs due to cholesterol deposition at the vessel wall and the formation of atherosclerotic plaques. This plaque may rupture and lead to clot formation due to platelet activation, possibly closing the vessel completely, causing a heart attack^15^. The nutritional and medicinal value of honey combined with the presence of oligosaccharides has projected honey as a functional additive in fermented milk^16^. In addition, it is used as a sweetener and as a preservative in dairy products^17^. Research also indicates that honey-based kefir beverages are characterized by high antioxidant activity^18^. Furthermore, adequate amounts of honey do not negatively affect bacterial growth; for example, *Streptococcus thermophilus*, *Lactobacillus acidophilus*, *Lactobacillus delbrueckii* subsp. *bulgaricus* and *Bifidobacterium bifidum*^19^. According to Varga, the addition of 1–5% (w/v) honey had no significant effect on the viability of *Streptococcus thermophilus* and *Lactobacillus delbrueckii* subsp. *bulgaricus* during cold storage of yogurt. Furthermore, they found that addition of approx. 3% (w/v) of honey significantly improves the sensory quality of the finished product^10^. However, when Sert et al. examined the addition of 2%, 4%, and 6% (w/v) of sunflower honey, they showed that its presence in yogurt influences the growth and liveliness of *Lactobacillus delbrueckii* subsp. *bulgaricus* during the incubation and storage period of yogurt samples^19^. Coskun and Karabulut Dirican analyzed yogurt with addition of 2%, 4%, and 6% of pine honey, where the number of *Lactobacillus delbrueckii* subsp. *bulgaricus* and *Lactobacillus acidophilus* decreased and the number of *Streptococcus thermophilus* increased compared to the control sample without honey addition. However, the numbers of *Lactobacillus delbrueckii* subsp. *bulgaricus* and *Lactobacillus acidophilus* were above the recommended minimum number (≥ 10^6^ cfu/g)^17^. *Lactobacillus* bacteria are considered to be organisms with high antimicrobial and probiotic potential^20^. Păucean et al. showed that the addition of acacia honey in kefir production at 1%, 2.5%, and 4% (w/v) had no effect on the growth and viability of *Lactococcus* bacteria. Moreover, the same authors proved that the addition of honey reduces syneresis and increases the consistency of kefir, and no significant effect on pH and lactic acid production was shown^21^. Compared to blossom honeys, honeydew honey possesses potential health-promoting value due to its high bioactive compound content, including phenolics, proteins, and amino acids^22^. The growing market for this honey type significantly contributes to its increased price; however, consumers increasingly appreciate the taste and nutritional value of honeydew honey^23^. According to the literature reports, honey is mainly added to yogurt^10,16,17^. The addition of honey, especially honeydew honey to kefir, is not commonly described in the literature. Therefore, we combined the two products together: kefir and honeydew honey. We decided to take this step also, because most of the research on fortified kefir-based beverages focuses mostly on introducing vegetables as a source of bioactive ingredients. Our observations are confirmed by the review of the literature Aiello et al. The authors also noted the use of plant extracts and essential oils as a source of bioactive molecules in the production of kefir^6^. However, Du and Myracle described the possibilities of using aronia and elderberry in the production of kefir and thus obtaining sensory-acceptable functional food^24^. Similar scientific reports also suggest the use of blueberry and blackberry in the production of fermented beverages^25^.

In view of the above—the high pro-health value of kefir and honey prompts us to research the quality, mainly syneresis, texture and color resulting from the holding of water in the product. Awareness of the combination of kefir and honeydew honey of different densities may result in delamination of the mixture, which in turn may be reflected in the consumer acceptability of such a product. This work focuses on analyzing how the addition of honeydew honey and different compositions of starter cultures shape the sensory acceptability related to water activity, color, syneresis and consistency of the produced kefir because there are still not enough scientific reports on this topic. The presented work will show whether it is possible to create honeydew honey kefir with acceptable properties, which may have importance for further use in public health.

## Materials and methods

### Model and commercial kefir samples

Two different samples of kefir were examined. The samples did not differ (*p* > 0.05) in terms of the content of nonfat dry matter (116.0 g/kg), fat (20.0 g/kg), protein (34.0 g/kg), or pH (4.45), which was confirmed in our preliminary research. We performed preliminary studies during the design of the experiment. Kefirs differed by type of used starter cultures and conditions of production. The first sample of model kefir (K) was produced on a pilot plant scale using factory-scale equipment and was made using 6 bacterial strains: *Lactococcus lactis* subsp. *lactis*, *Lactococcus lactis* subsp. *cremoris*, *Lactococcus lactis* subsp. *lactis biovar diacetylactis*, *Levilactobacillus brevis*, *Leuconostoc* and yeasts *Saccharomyces cerevisiae* as starter cultures, which were commercially available from Lyofast MT 032 LV (Sacco, Cadorago, Italy), and added at 0.25 units per 25/L processed milk. Fermentation ran at 26 °C until pH 4.45 was reached. A two-step cooling to 15 °C for a maximum of 15 min was applied, and the product was poured into unit containers of v = 1 L and further cooled to 6 °C. The second sample of commercial kefir (K13) was a commercial product with 13 bacterial strains: *Bifidobacterium lactis, Bifidobacterium infantis, Lactobacillus acidophilus, Lactobacillus delbrueckii* subsp*. lactis, Limosilactobacillus fermentum, Lacticaseibacillus paracasei, Lacticaseibacillus rhamnosus, Lactococcus lactis* subsp*. cremoris, Lactococcus lactis* subsp*. lactis biovar diacetylactis, Lactococcus lactis* subsp*. lactis, Leuconostoc mesenteroides, Leuconostoc pseudomesenteroides, Streptococcus thermophilus* and yeasts *Debaryomyces hansenii* (OSM, Koło, Poland). It was the newest product available to consumers in the local market. Additionally, the study involved the possibility of kefir preparation from the dairy industry with honey by consumers at home.

Bacteria and yeasts were enumerated in the model and commercial kefir by plating 500 mL of each diluted sample on appropriate agar media as described by Nambou et al.^26^. Our preliminary research showed that mesophilic LAB in kefir K and K13 were 6.48 and 6.35 log cfu/mL, respectively, and those in yeasts were 4.53 and 4.62 log cfu/mL, respectively. Cell counts of different microorganisms present in model and commercial kefir meet the requirements of Codex Alimentarius^27^. Preliminary research also included the determination of antioxidant potential expressed as the ability of an antioxidant to scavenge stable 1,1-diphenyl-2-picrylhydrazyl (DPPH) according to the method described by Bierzuńska and Cais-Sokolińska^28^. The antioxidant potentials of samples K and K13 were 2435.0 and 2622.0 µmol Trolox/kg, respectively. In addition, there was no effect of sample type, amount of added honeydew honey or storage time on antioxidant potential.

### Honey

Honeydew honey from silver fir (over 78%, the rest is spruce and pine) was produced at an apiary in the Podkarpacie region (Lubaczów district, Poland). Honey was purchased at the market during a local festival directly from beekeepers. The honey came from an apiary with Carniolan honey bees (*Apis mellifera*)*.* In the preliminary study, the composition and characteristics of honey were determined. The main components of honey were as follows: water 167.0 g/kg, glucose 272.0 g/kg; fructose 337.0 g/kg, sucrose 51.0 g/kg, maltose 21.0 g/kg, trechalose 1.0 g/kg, melecytose 4.0 g/kg, HMF 0.34 mg/kg, minerals 8.0 g/kg, protein 6.0 g/kg, phenolic acid, and p-coumaric acid 26.13 mg/100 g. Properties: electrical conductivity 9.2 × 10^−4^ S/cm, pH 4.31, antioxidant activity 1620.34 µmol Trolox/kg, total phenolic compounds 301.22 mg gallic acid/kg, vitamin C 4.3 mg/100 g. Honey was no more than 2 months old and stored in glass jars (v = 250 mL) at room temperature in a dark, dry place.

### Experimental design

Kefir was combined with honey no later than the second day after manufacturing (n = 8). Kefir was kept at 6 °C and honey at 18 °C. Mixing was carried out for 10 s using a mechanical stirrer (RPM 20, MRK-12, MPM, Milanówek, Poland). Each sample (v = 1 L) kefir (K and K13) was supplemented with 2.5% and 5% honeydew honey (w/w), and there were 6 samples (including 4 samples with honey). The samples were tested after the completion of mixing (day 0) and at 14 days of cold storage, i.e., at 6 °C ± 0.5 °C.

### Determination of water holding capacity

The water holding capacity (WHC) of kefir is defined as its ability to hold all or part of its own water. The WHC of the kefir samples was determined using the centrifugation method^29^. Kefir (30 g) was centrifuged (model 260; MPW MED Instruments, Warsaw, Poland) under relative centrifugal force (RCF) = 10 732 g, 30° (RPM 10 062 g) at 4 °C for 15 min. The supernatant was collected and weighed, and WHC was calculated according to Eq. 1:

1 $${\text{WHC}}\; \, \left( \% \right) \, = \, \left( {1 \, {-}{\text{ W}}_{1} /{\text{W}}_{2} } \right) \, \cdot \, 100$$

where W_1_ is the weight in grams of the supernatant after centrifugation and W_2_ is the weight of kefir in grams.

### Spontaneous whey syneresis

The siphon method described by Bierzuńska et al. was employed in the study. A cup of kefir (100 mL) was tilted 45° immediately after being removed from the refrigerator to collect the surface whey; this was siphoned out using a graduated syringe with a needle attached. Siphoning was performed within 10 s to avoid forced leakage of whey from curd^30^.

### Water activity

The water activity was measured using an AquaLab Series 4TE instrument (Decagon Devices Inc., Pullman, USA) based on p_f_ (T), the value of the water vapor that was in equilibrium with the sample maintained at a constant level during the measurement at temperature T, and p_s_ (T), the vapor pressure of saturated pure water at the same temperature T, as a_w_ = p_f_ (T) ∙ p_s_ (T)^−1^. Samples of v = 15 mL provided were placed in DE 501 measurement vessel DE 501 vessels (Decagon Devices Inc., Pullman, USA) and tested at 15 °C.

### Profile texture analyses

The firmness, consistency, cohesiveness and viscosity index of the fermented kefir samples were determined using reverse extrusion using a TA-XTplus texturemeter from Stable Micro Systems (Surrey, UK)^31^. A/BE attachment with a compression disc (Ø = 35 mm) was used. A sample was placed inside a cylinder with an internal diameter Ø = 50 mm (75% filling) at a distance of 30 mm, pretest 1.0 mm/s and posttest 10.0 mm/s. The results were recorded using Texture Exponent E32 version 4.0.9.0 software (Godalming, Surrey, UK).

### Color

The instrumental color measurement was based on the CIELab system described by Cais-Sokolińska et al.^32^. The measurement was performed with geometry SPIN using an X-Rite SP-60 camera (Grandville, MI, USA) equipped with a spherical geometry (diffusive) and the measurement chamber with a DRS-811 ceramic insert. The camera was calibrated based on the white and black reference standards SP-62-162 (Grandville, MI, USA). The chrome (C*) (Eq. 2), white index (WI) (Eq. 3), yellowing index (YI) (Eq. 4) were calculated using the following equations:

2 $${\text{C}}* \, = \, \left[ {\left( {\Delta {\text{a}}*} \right)^{2} + \, \left( {\Delta {\text{b}}*} \right)^{2} } \right]^{0.5}$$

3 $${\text{WI }} = \, \left[ {\left( {\Delta {\text{L}}} \right)^{2} + \, \left( {\Delta {\text{a}}*} \right)^{2} + \, \left( {\Delta {\text{b}}*} \right)^{2} } \right]^{0.5}$$

4 $${\text{YI }} = \, 142.86{\text{b}}* \, \cdot{\text{ L}}^{ - 1}$$

For the calculations, it was assumed that L = 100, a* = 0 and b* = 0.

### Sensory analysis

Sensory analysis was conducted via the profiling method^33,34^. Panelists: Thirteen people aged between 20 and 54 were adequately trained individuals prepared to perform sensor examinations^35,36^. Samples were evaluated using 8 cm unstructured line scales anchored with the terms low (denotes an undetectable points parameters) at the left and high (very intense) at the right. Sample temperature was 10–12 °C. The descriptors are listed in Table 1.

**Table 1:** Sensory attributes and description used to characterize kefir with honey.

Attribute type and attributes	Description	Reference
*Taste*		
Sweet	Taste sensation associated with sugars	1—Kefir; 9—honeydew honey
Sour	Taste sensation associated with acids	1—Sweet milk; 9—kefir
*Flavor*		
Astringent	Chemical feeling factor associated with the shrinking and puckering of tongue	1—Kefir; 9—honeydew honey
Fruity	Flavor associated with fruity flavors	1—Kefir; 9—honeydew honey
İnvigorating	Flavor associated with a refreshing drink	1—Pasteurized milk; 9—kefir
Kefir-like	Complex olfactory sensation due to fermentation of milk with kefir cultures	1—Pasteurized milk; 9—kefir
Milky	Aromatics associated with fresh pasteurized milk	1—Kefir; 9—pasteurized milk 2% fat
Pungent	Flavor associated with a refreshingly pungent smell	1—Kefir; 9—honeydew honey
Waxy	Aromatics associated with coniferous resin fragrance	1—Kefir; 9—honeydew honey
Yeast	Flavor associated with fermenting yeast	1—Pasteurized milk; 9—kefir
*Texture and mouthfeel*		
Creaminess	Velvety or soft feeling in the mouth (not fatty or oily)	1—Kefir; 9—UHT cream 35% fat
Density	Thickness of the samples in the mouth	1—Pasteurized milk; 9—kefir
Firmness	Perceived firmness of the sample evaluated in the mouth	1—Pasteurized milk; 9—kefir
Prickling	Tingling feeling on the tongue similar to a carbonated mineral water	1—Pasteurized milk; 9—kefir

### Statistical analyses

Verification of statistical hypotheses was achieved using a level of significance of α = 0.05. The influence of the composition and storage time on the samples was evaluated by two-way analysis of variance (one-way ANOVA) followed by Tukey’s HSD post hoc test for multiple comparisons. Data were analyzed using Statistica data analysis software, version 13 (TIBCO Software Inc., Palo Alto, California, USA).

### Ethical statement

All people participating in the sensory analysis given their informed consent to participate. All methods were carried out in accordance with relevant guidelines and regulations. According to Polish law and GCP regulations, this research does not require approval of the Bioethics Committee and was not a medical experiment. Confirmation was issued by the Bioethics Committee at Poznan University of Medical Sciences (number of decisions is KB-332/21).

## Results and discussion

### Acidity, activity and mobility of water and water holding capacity in kefir with honeydew honey

Analysis of the physicochemical properties showed no statistically significant differences in acidity in the K and K13 controls, as shown in Table 2 (*p* > 0.05). The addition of honey to kefir increases acidity by approx. 17.6% in samples model kefir with 2.5% honeydew honey (K_2.5) and model kefir with 5.0% honeydew honey (K_5.0), and by approx. 10% in commercial kefir with 2.5% honeydew honey (K13_2.5) and commercial kefir with 5.0% honeydew honey (K13_5.0) (*p* < 0.05). The addition of honey (regardless of the quantity) only to the model kefir did not change the acidity during storage (*p* > 0.05).In commercial kefir (regardless of the amount of honeydew honey introduced), the acidity decreased by approx. 7% during storage (*p* < 0.05). Elenany, who analyzed goat milk yogurt with the addition of marjoram honey, found that the increase in acidity of fermented milk may be related to the presence of prebiotic oligosaccharides in honey which may promote the growth and the metabolic activity of lactic acid bacteria^37^.

**Table 2:** Changes in physicochemical properties of kefir with honeydew honey during storage.

	Storage (d)	Sample						SEM
		K	K_2.5	K_5.0	K13	K13_2.5	K13_5.0	
pH	0	4.22^aA^	4.18^aA^	4.18^aA^	4.49^bA^	4.35^abA^	4.37^abA^	0.007
	14	4.27^bB^	4.23^aB^	4.23^aB^	4.48^dA^	4.45^cB^	4.46^cdB^	0.000
Acidity (°SH)	0	32.4^aA^	38.1^cA^	38.0^cA^	32.0^aA^	35.1^bB^	35.2^bB^	0.056
	14	37.8^bB^	37.8^bA^	37.6^bA^	32.1^aA^	32.6^aA^	32.6^aA^	0.682
SWS (%)	0	0.2^abA^	0.4^bA^	0.4^bA^	0.1^aA^	0.3^abA^	0.4^bA^	0.004
	14	3.8^eB^	3.2^ dB^	2.7^cB^	2.6^cB^	1.2^bB^	0.8^aB^	0.014
WHC (%)	0	78.09^aB^	79.05^aA^	78.40^aA^	89.24^bB^	90.88^bB^	95.09^cB^	0.568
	14	71.60^aA^	79.31^bA^	79.49^bA^	81.71^bA^	89.20^cA^	91.34^cA^	0.791
Water activity	0	0.9742^bA^	0.9699^bB^	0.9591^aA^	0.9715^bA^	0.9630^aA^	0.9610^aB^	0.000
	14	0.9747^dA^	0.9662^bcA^	0.9650^bB^	0.9711^cdA^	0.9662^bcA^	0.9562^aA^	0.000

K, model kefir; K13, commercial kefir; K_2.5, model kefir with 2.5% honeydew honey; K_5.0, model kefir with 5.0% honeydew honey; K13_2.5, commercial kefir with 2.5% honeydew honey; K13_5.0, commercial kefir with 5.0% honeydew honey; d, day; SWS, spontaneous whey syneresis; WHC, water holding capacity; SEM, standard error of the mean (n = 8).

^a–e, A–B^ Different lowercase letters in the superscript in rows and capital letters in columns for each parameter indicate statistically significant differences at the level α = 0.05.

The disadvantage of sensory attractiveness of fermented milk is syneresis, which is a consequence of shrinkage of milk protein gel, which decreases the size of casein aggregates and leads to the separation of whey^38^. Whey separation is affected by various factors (pH, acidity, total solids, hydrocolloid content, etc.). Syneresis is the expulsion of whey from three-dimensional networks, which become visible on the surface, which affects the abbreviation shelf life of fermented milk due to changes in appearance and texture^39^.

The results in Table 2 show that the addition of honey to kefir causes a twofold increase in spontaneous whey syneresis (SWS) in samples K_2.5 and K_5.0 compared to sample K and a fourfold increase in the case of kefir K13_5.0 compared to sample K13 (*p* < 0.05). However, only a twofold increase in SWS during storage was observed in K13_5.0. It possesses the highest water holding capacity (WHC), with a 21.8% higher value than K. According to Sert et al., this is related to the fructose content of honey, which is capable of binding to water^19^. However, only in the K_2.5 and K_5.0 kefirs were there no statistically significant differences in WHC during storage (*p* > 0.05). The addition of 5% honey, regardless of the starter culture used, reduced WHC (*p* < 0.05).

### Texture and color of kefir with honeydew honey

Rheological property analysis is important in the determination of the various interactions in kefir samples. Maintaining the proper texture of fermented milk can be challenging in the commercial manufacturing of alternative fermented dairy products^40^. In this study, as shown in Table 3, no statistically significant differences were found in the analysis of firmness, consistency and cohesiveness in kefir produced by *Lactococcus lactis* subsp. *lactis*, *Lactococcus lactis* subsp. *cremoris*, *Lactococcus lactis* subsp. *lactis biovar diacetylactis*, *Levilactobacillus brevis*, *Leuconostoc* and yeast *Saccharomyces cerevisiae* regardless of the additive used (*p* > 0.05). Kefir produced using 13 strains of bacteria and yeast exhibited greater firmness by 22.4% and 4.8-fold higher viscosity index compared to sample K (*p* < 0.05). K_2.5 shows increased parameter properties, including firmness, consistency and viscosity index texture, of 6.9%, 7.8% and 26.3%, respectively (*p* < 0.05). However, no variances were found for K_5 (*p* > 0.05). There were no statistically significant differences in firmness, consistency or viscosity index during the cold storage of model kefir (K) with the addition of honeydew honey (*p* > 0.05). The texture of fermented milk is of great importance for the acceptability of the product, therefore it should be stable throughout its storage period^41^. According to Păucean et al., the addition of honey may increase the perceived viscosity of the samples because its addition can promote greater total solids concentrations^21^. However, according to Mohan et al.’s texture parameters, for example, firmness was positively correlated with the total solids^42^. The texture of kefir is a reflection of the ratio of casein to whey protein fractions and the size of casein micelles in the milk being fermented. Casein proteins are found in milk as micelles that form a colloidal solution. Micelles consist of monomers of individual casein fractions linked together by bridges formed by calcium, phosphate and citrate ions. On average, there are approximately 7·10^13^ micelles in 1 cm^3^ of milk, and their diameter significantly affects the measure of internal friction, that is, viscosity. In cow's milk, the proportion of the α_s1_-casein fraction affecting the consistency is 48.5% of the total casein protein (25 g/kg)^43^.

**Table 3:** Changes in texture parameters in kefir with honeydew honey during storage.

Texture parameters	Storage (d)	Sample						SEM
		K	K_2.5	K_5.0	K13	K13_2.5	K13_5.0	
Firmness (g)	0	21.76^aA^	21.43^aA^	21.02^aA^	26.64^bA^	28.48^cA^	27.07^bA^	0.196
	14	22.43^aB^	21.90^aA^	20.90^aA^	27.69^bB^	31.62^cB^	30.69^cB^	0.544
Consistency (g∙s)	0	489.44^aA^	480.47^aA^	474.10^aA^	629.42^bA^	678.50^cA^	632.38^bA^	95.351
	14	507.06^bB^	494.08^abA^	465.94^aA^	650.84^cB^	749.99^eB^	711.81^ dB^	172.880
Cohesiveness |(g)|	0	13.76^aB^	13.60^aB^	12.71^aA^	18.48^bA^	19.88^bA^	18.31^bA^	0.432
	14	11.90^aA^	12.55^aA^	12.21^aA^	18.60^bA^	20.05^bA^	18.76^bA^	0.691
Viscosity index |(g∙s)|	0	5.05^bB^	2.60^aA^	1.34^aA^	24.04^cA^	30.36^dA^	24.32^cA^	0.499
	14	1.59^aA^	0.20^aA^	0.45^aA^	26.83^bB^	33.83^cB^	27.33^bB^	4.305

K, model kefir; K13, commercial kefir; K_2.5, model kefir with 2.5% honeydew honey; K_5.0, model kefir with 5.0% honeydew honey; K13_2.5, commercial kefir with 2.5% honeydew honey; K13_5.0, commercial kefir with 5.0% honeydew honey; d, day; SEM, standard error of the mean (n = 8).

^a–e, A–B^ Different lowercase letters in the superscript in rows and capital letters in columns for each parameter indicate statistically significant differences at the level α = 0.05.

The addition of 5% honey reduced the brightness parameter (L*) by 4.7% and 5.3% compared to the control samples K and K13, respectively, as shown in Table 4 (*p* < 0.05). In addition, the greatest white index was observed, which was independent of the starter culture used in the production of kefir (*p* < 0.05). 2.5% honey did not change the chrome C* (*p* > 0.05). During cold storage, no parameter changes in the brightness L* and white index WI in the model kefir with the addition of 2.5% honey were detected (*p* > 0.05). Furthermore, no changes occurred in chrome C* during refrigerated storage in the K13_2.5 sample (*p* > 0.05). Dimitreli et al. examined inter alia, in which the effect of post fermentation addition of fir honey on the physicochemical, rheological and sensory characteristics of kefir showed reduced brightness parameter (L*) and red color intensity^8^. Color is an important determinant of quality and can affect consumer acceptability^42^. Color changes are possible at all stages of milk processing, e.g., the Maillard reaction during heating^44^. The color change is also related to the fermentation process. Therefore, instrumental color measurement becomes important^45^. Moreover, based on the color measurement, optimization and selection of the technological process conditions can be made^30^.

**Table 4:** Change assessment of the color of kefir with honeydew honey during storage.

Assessment of color	Storage (d)	Sample						SEM
		K	K_2.5	K_5.0	K13	K13_2.5	K13_5.0	
L*	0	90.51^cB^	89.20^bA^	86.29^aB^	94.38^eB^	92.18^ dB^	89.39^bA^	0.139
	14	85.81^aA^	87.01^aA^	85.35^aA^	92.68^bA^	90.69^bA^	91.29^bA^	1.479
WI	0	11.97^cA^	12.79^cA^	14.92^dA^	9.07^aA^	10.41^bA^	12.67^cB^	0.103
	14	15.26^cB^	14.22^bcA^	15.96^cB^	9.92^aA^	11.53^abB^	10.78^aA^	1.115
YI	0	11.10^cB^	10.48^bcB^	9.33^aA^	10.23^bA^	10.12^bA^	10.42^bcB^	0.072
	14	8.89^aA^	8.94^aA^	10.25^cB^	9.85^bcA^	10.23^cA^	9.53^bA^	0.026
C*	0	7.30^bB^	6.84^bB^	5.91^aA^	7.11^bB^	6.87^bA^	6.91^bB^	0.035
	14	5.60^aA^	5.69^aA^	6.33^bB^	6.70^cA^	6.80^cA^	6.32^bA^	0.013

K, model kefir; K13, commercial kefir; K_2.5, model kefir with 2.5% honeydew honey; K_5.0, model kefir with 5.0% honeydew honey; K13_2.5, commercial kefir with 2.5% honeydew honey; K13_5.0, commercial kefir with 5.0% honeydew honey; d, day; L*, brightness; WI, white index; C*, chrome; YI, yellowing index; SEM, standard error of the mean (n = 8).

^a–e, A–B^ Different lowercase letters in the superscript in rows and capital letters in columns for each parameter indicate statistically significant differences at the level α = 0.05.

### Sensory analysis of kefir with honeydew honey

**Figure 1:**
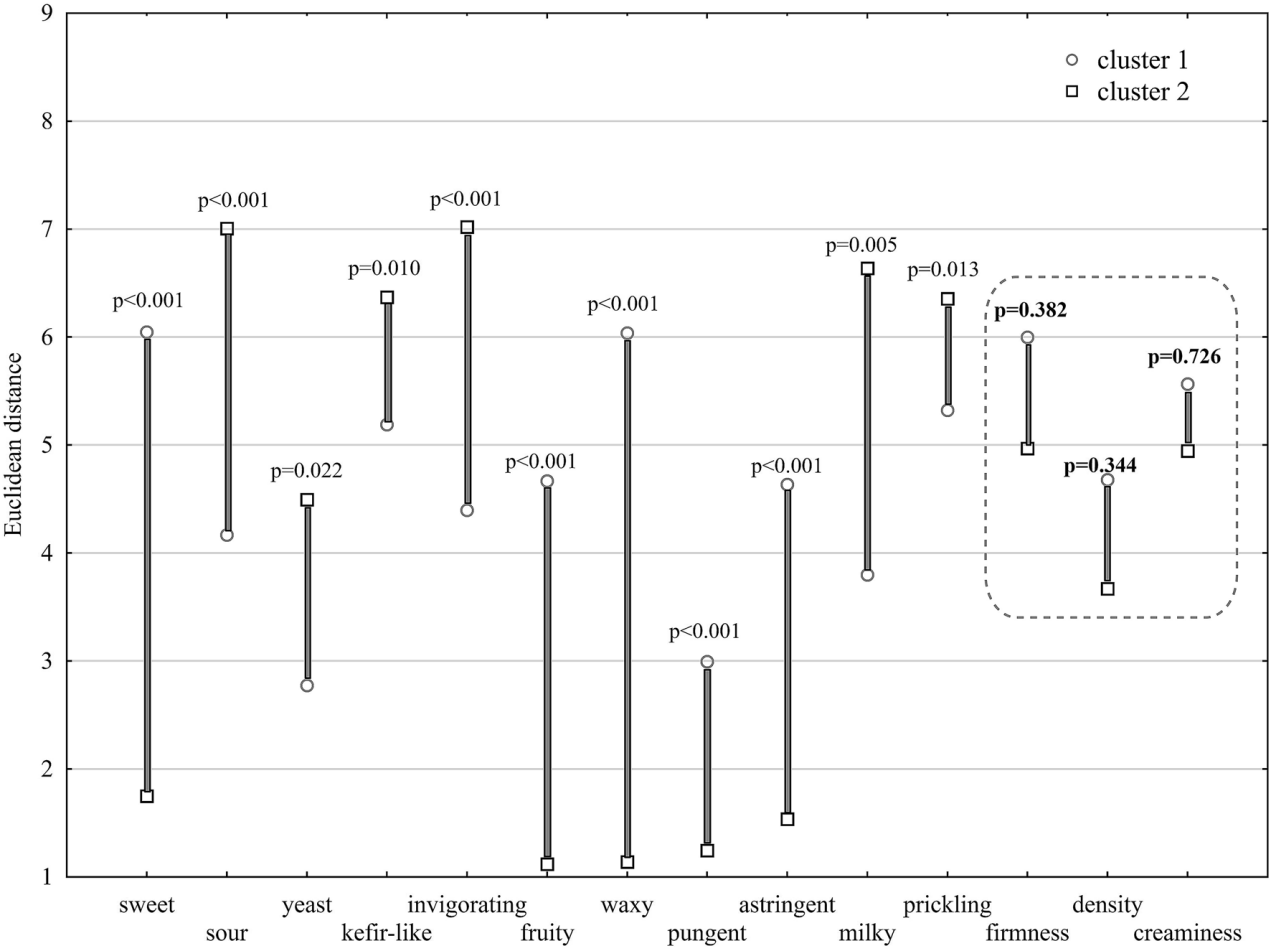
Grouping by the k-means of sensory profiling of kefir with the addition of honey in different amounts and storage times.

**Figure 2:**
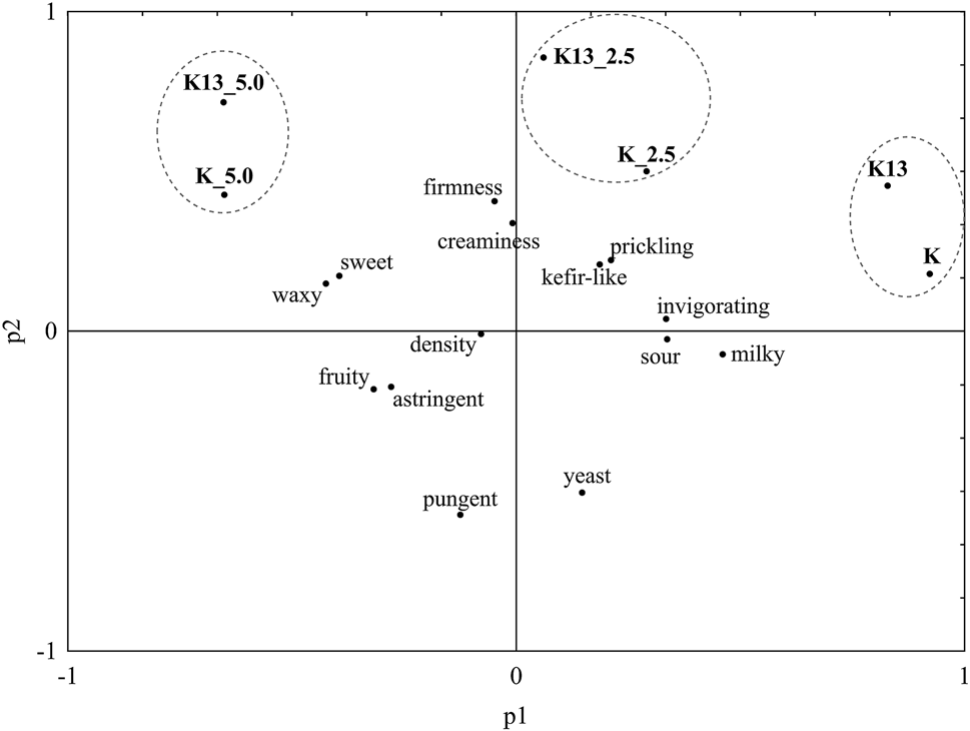
Principal component analysis biplot of sensory parameters used to differentiate kefir honeydew honey. K, model kefir; K13, commercial kefir; K_2.5, model kefir with 2.5% honeydew honey; K_5.0, model kefir with 5.0% honeydew honey; K13_2.5, commercial kefir with 2.5% honeydew honey; K13_5.0, commercial kefir with 5.0% honeydew honey.

Reducing the mean values of each sample at different storage times to one dimension resulted in only two clusters. K-means cluster analysis showed that attributes of texture and mouthfeel, creaminess (F = 0.130, *p* = 0.726), density (F = 0.986, *p* = 0.344) and firmness (F = 0.836, *p* = 0.382), had no significance in relation to the refrigerated storage time and added honey, as shown in Fig. 1. The most important parameters of kefir with honeydew honey are the attributes of flavor: astringent, fruity, pungent and waxy due to the longest Euclidean values. These observations were confirmed by principal component analysis (PCA), as shown in Fig. 2. The results show that as honey content increases, kefir has more fruit and astringent flavors. The difference between the addition of 2.5% and 5.0% of honey is not significant. However, compared to kefir without the addition of honeydew honey, the flavor pungent is 3 times more perceptible. Regardless of the starter culture used, the addition of 2.5% honeydew honey had no influence on flavor, including invigorating and kefir-like flavors, or attributes of texture and mouthfeel: prickling. The addition of 5% honeydew honey to kefir can be characterized by attributes of taste (sweet) and flavor (waxy). It is presumed that the sweet and waxy flavor may have the greatest impact on the assessment of their acceptability by consumers. Samples K_5.0 and K13_5.0 have sweet tastes with scores of 6.8 and 7.7 on a scale of 1–9, respectively, making the samples more like honey than kefir. Additionally, high marks are obtained for the waxy flavor of the samples K_5.0 and K13_5.0 (6.5 and 7.7 score, respectively). Hence, the supposition that the addition of 5% may be too high and that the addition of honey to kefir in the amount of 2.5% is more beneficial with consumers in various age groups.

According to Larosa et. al use of honey for kefir improves acceptance in appearance and aroma^46^. Păucean et al. showed that the optimal addition of honey to kefir was 2.5% (w/v), and the introduction of 4% honey (w/v) made the kefir too sweet. Additionally, they found that improvement of syneresis, consistency, taste and flavor can be obtained with 2.5% honey, and the production of kefir with the addition of honey may be an alternative for desired taste and nutrition for new fermented dairy beverages^21^. However, Mohan et al. showed that the introduction of 5% (w/v) Manuka honey to yogurt can enhance both the functional health value and consumer acceptance^42^.

## Conclusions

The addition of honeydew honey to fermented milk causes greater WHC and reduces water activity, regardless of the type of starter culture used. The use of a different starter culture in kefir production with the addition of honeydew honey has an impact on the texture parameters, resulting in up to a 4.89-fold increase in the viscosity index. Measuring the parameters of texture, color, and syneresis of fermented milk with honey is important due to the development of its quality and associated consumer acceptability. Implementing honeydew honey into kefir is a novel food example. At the same time, the entire technological protocol of kefir with honey was developed. As a result of combining the well-known kefir and honey, it is possible to obtain a product with completely new properties and enriched value, which may be a response to the new needs of the consumers. This is important due to the growing awareness of the consumer about the relationship between diet and health.
